# The pontine diffuse midline glioma, *EGFR*‐subtype with ependymal features: Yet another face of diffuse midline glioma, H3K27‐altered

**DOI:** 10.1111/bpa.13181

**Published:** 2023-06-18

**Authors:** Arnault Tauziède‐Espariat, Alice Métais, Cassandra Mariet, David Castel, Jacques Grill, Raphaël Saffroy, Lauren Hasty, Volodia Dangouloff‐Ros, Nathalie Boddaert, Sandro Benichi, Fabrice Chrétien, Pascale Varlet

**Affiliations:** ^1^ Department of Neuropathology GHU Paris‐Psychiatrie et Neurosciences, Sainte‐Anne Hospital Paris France; ^2^ Inserm, UMR 1266, IMA‐Brain Institut de Psychiatrie et Neurosciences de Paris Paris France; ^3^ U981, Molecular Predictors and New Targets in Oncology, INSERM, Gustave Roussy Université Paris‐Saclay Villejuif France; ^4^ Department of Pediatric Oncology, Gustave Roussy Université Paris‐Saclay Villejuif France; ^5^ Department of Biochemistry and Oncogenetic Paul Brousse Hospital, APHP Villejuif France; ^6^ Pediatric Radiology Department Hôpital Necker Enfants Malades, AP‐HP Paris France; ^7^ UMR 1163, Institut Imagine and INSERM U1299 Université Paris Cité Paris France; ^8^ Department of Pediatric Neurosurgery, Necker Hospital, APHP Université Paris Descartes, Sorbonne Paris Cite Paris France

**Keywords:** diffuse midline glioma, EGFR, ependymoma, EZHIP

## Abstract

Diffuse midline glioma with two components: classical glial and ependymal.

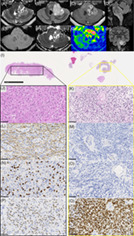

In the central nervous system, both posterior fossa A ependymomas (PFA‐EPNs) and diffuse midline gliomas (DMGs), H3K27‐altered feature a loss of H3K27me3 immunoexpression. These entities have been recently divided into four different molecular subtypes: H3.3‐mutant, H3.1‐mutant, H3‐wildtype with EZHIP overexpression, and *EGFR*‐mutant (with the last two having an overexpression of EZHIP). While PFA‐EPN and DMG, H3K27‐altered both have a pediatric onset and share molecular features (histone gene mutations or EZHIP overexpression), they are fundamentally distinguished by their ability to diffusely infiltrate. Moreover, a potential morphological overlap may exist between these two glial tumor types. For example, an initially diagnosed DMG, EZHIP‐overexpressing of the brainstem was later reclassified as a PFA‐EPN using DNA‐methylation analysis [1], and reciprocally, DMG, H3K27M‐mutant having ependymal features have been described [2]. Herein, we report for the first time a DMG, *EGFR*‐mutant with ependymal features.

This case concerned an 11‐year‐old girl who presented symptoms over a period of 3 months. She began with a peripheral facial palsy, then experienced diplopia, dysphagia, and an ataxia revealing a pontine tumor. Radiologically, the tumor was intrinsic to the pons, infiltrative, and nodular (Figure 1A–H). Histopathologically, this tumor was biphasic, composed of two distinct glial components: one solid with ependymal features without Olig2 expression and one infiltrative glial component having Olig2 expression (Figure 1I–O). Both components presented a loss of H3K27me3 and expressed EZHIP (Figure 1P,Q) (without H3K27M and EGFR immunopositivities). A *TP53* mutation was found by DNA sequencing analysis, without mutation of *HIST1H3B*, *HIST1H3C*, *H3F3A*, or *ACVR1*. No *EGFR* mutation or amplification was evidenced. After microdissection, the two components were analyzed using DNA‐methylation profiling, and a low calibrated score (0.45 and 0.31) for DMG‐EGFR (v12.5 of the brain classifier) was proposed but both clustered using the t‐distributed stochastic neighbor embedding (t‐SNE) analysis in close vicinity to the DMG‐EGFR MC cluster (Figure 2). Following the biopsy, the patient received radiation therapy, chemotherapy with Everolimus, and was alive without progression 8 months after the onset of the symptoms with a stable disease. The integrated diagnosis of this case was DMG, H3 K27‐altered (a possible *EGFR* subtype) with ependymal features.

**FIGURE 1:**
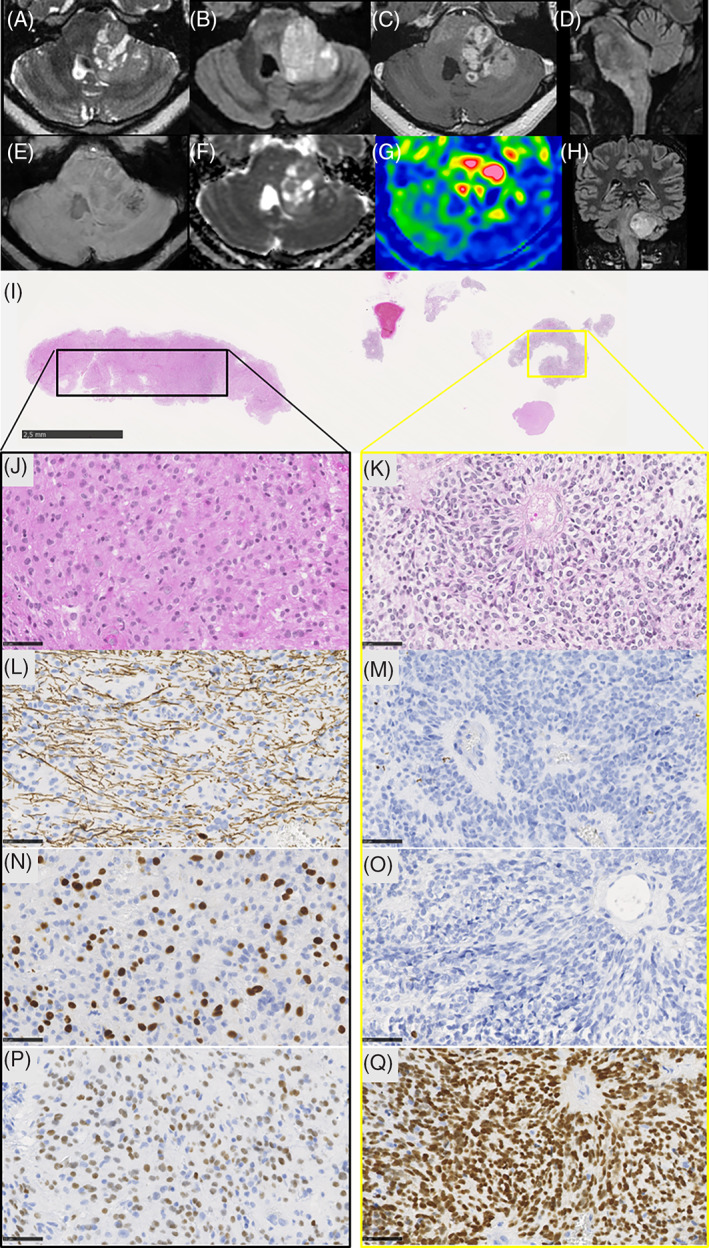
Radiological and histopathological features of the case. Axial (A–C, E–G), sagittal (D), and coronal (H) images centered on the posterior fossa. (A) T2‐weighted image, (B, D, H) FLAIR images, (C) T1‐weighted image after gadolinium injection, (E) susceptibility‐weighted images, (F) apparent diffusion coefficient map, (G) arterial spin labeling cerebral blood flow map. Images show a tumor with two components: one bulky component centered in the left middle cerebellar peduncle, and one adjacent infiltrative component centered in the pons, with an extension toward the medulla oblongata. The bulky part showed solid and cystic content, with strong contrast enhancement, little hemorrhage, partially restricted diffusion, and high cerebral blood flow. The infiltrative part showed a heterogeneous, high FLAIR signal, faint contrast enhancement, no hemorrhage or diffusion restriction, and intermediate cerebral blood flow. The biopsy highlighted two different histopathological components (I, hematoxylin phloxin saffron [HPS], magnification 10×). The first component (J, L, N, P) was glial (J, HPS, magnification 400×), with a diffuse pattern using neurofilament immunostaining (L, magnification 400×), with a diffuse immunopositivity for Olig2 (N, magnification 400×), and diffuse immunoexpression of EZHIP (P, magnification 400×). The second component (K, M, O, Q) was ependymal with rosettes (K, HPS, magnification 400×), a circumscribed pattern using neurofilament immunostaining (M, magnification 400×), without expression of Olig2 (O, magnification 400×), but with a similar diffuse immunoexpression of EZHIP (Q, magnification 400×). Scale bars represent 2.5 mm (I) and 50 μm (J–Q).

**FIGURE 2:**
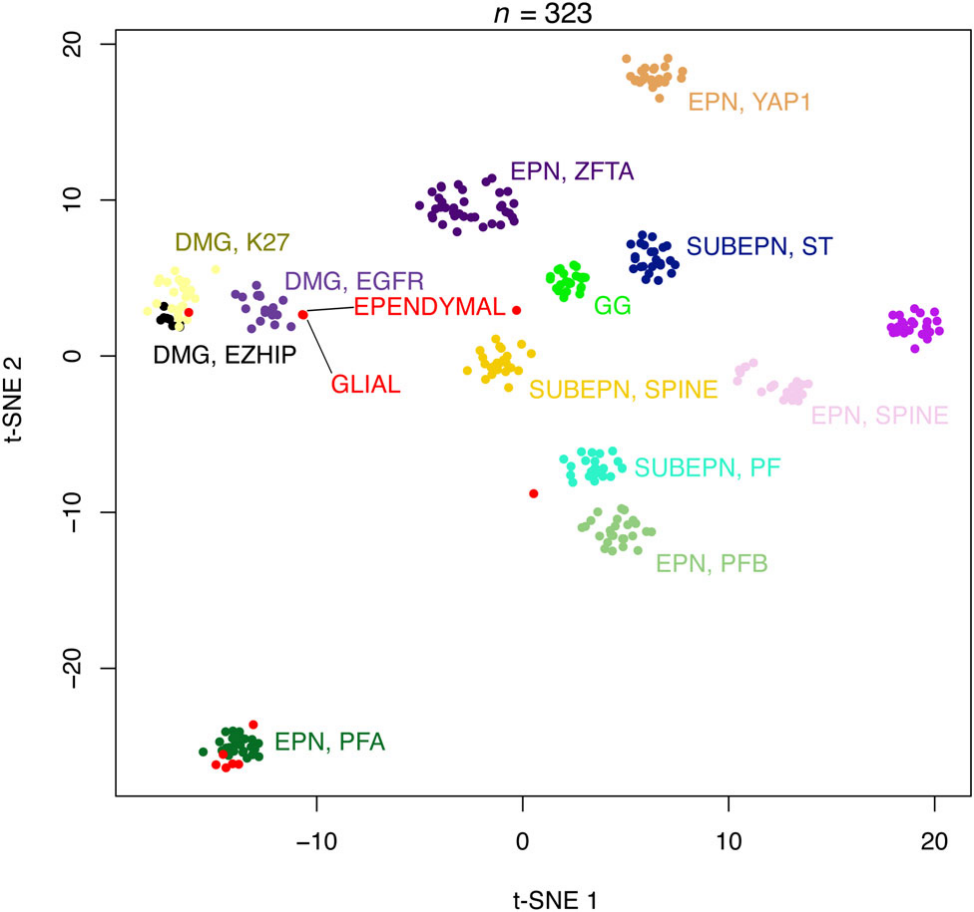
t‐Distributed stochastic neighbor embedding (t‐SNE) plot analysis of the current case with the two components (glial and ependymal) compared with reference samples from the Heidelberg cohort. DMG, EGFR: diffuse midline glioma, *EGFR*‐altered; DMG, EZHIP: diffuse midline glioma, EZHIP‐overexpressing; DMG, K27: diffuse midline glioma, H3K27M‐mutant; EPN, PFA: ependymoma of the posterior fossa, subtype A; EPN, PFB: ependymoma of the posterior fossa, subtype B; EPN, SPINE: ependymoma of the spine; EPN, YAP1: ependymoma, *YAP1*‐fusion positive; EPN, ZFTA: ependymoma, *ZFTA‐*fusion positive; GG: ganglioglioma; SUBEPN, PF: subependymoma of the posterior fossa; SUBEPN, SPINE: subependymoma of the spine; SUBEPN, ST: supratentorial subependymoma. Previously reported cases [2] are designated in red, the current case is designated by black lines.

This novel case highlights the difficulties encountered when reaching a diagnosis and the potential overlaps with DMG, H3K27‐altered. Our observation confirms that DMG, H3K27‐altered may present morphological ependymal features distinct from PFA‐EPN, which have different enhancer signatures [3]. Despite its preferential bithalamic location, DMG, *EGFR*‐mutant may also be monothalamic, cerebellar, or located in the pontine region [4]. Along with the determination of EZHIP overexpression, numerous data must be obtained before proposing an integrative diagnosis. These include a precise location (intrapontic or not), Olig2 immunoexpression, diffuse architecture or not, molecular analysis (H3 status, *EGFR* mutation status or amplification, cooperating mutations [*TP53*, *PPM1D*, *ACVR1*, MAPKinase, and *PDGFRA*]) and also epigenetic data [4, 5]. Complexly, a subset of DMG, *EGFR*‐altered (20%, 8/40 cases) did not harbor *EGFR* alterations [4], like the current case. *TP53* mutations seem to be very rare in DMG‐EZHIP [6] but frequent in DMG‐EGFR and thus may prompt neuropathologists to seek out *EGFR* alterations for an accurate diagnosis. The current case shared clinical, neuroradiological (pediatric pontine tumor), and molecular similarities (*TP53* mutation without *EGFR* alteration) with one previously reported case (case #17 in [4]). For these two cases, the DNA‐methylation classification of both morphological components was the same, confirming that the epigenetic profile is not dominated by genetic alterations but possibly by the cell of origin. This subgroup of DMG‐H3K27‐altered (without *EGFR* alterations but epigenetically close to DMG‐EGFR) may perhaps represent a distinct subtype mislabeled by the current classifier. For these reasons, further cases with ependymal features are needed to compare their genetic, epigenetic, clinical, and prognostic characteristics to classical DMG, H3K27‐altered.

In summary, DMG‐H3K27‐altered with ependymal features may constitute a morphological pitfall of PFA‐EPN. This challenging diagnosis integrates radiological, histopathological, genetic, and epigenetic data.

## AUTHOR CONTRIBUTIONS

Arnault Tauziède‐Espariat, Cassandra Mariet, Jacques Grill, Sandro Benichi, Volodia Dangouloff‐Ros, and Nathalie Boddaert compiled the MRI and clinical records. Arnault Tauziède‐Espariat, Alice Métais, Fabrice Chrétien, and Pascale Varlet conducted the neuropathological examinations. Arnault Tauziède‐Espariat, David Castel, and Raphaël Saffroy conducted the molecular studies. Arnault Tauziède‐Espariat, Lauren Hasty, and Pascale Varlet drafted the manuscript. All authors reviewed the manuscript.

## CONFLICT OF INTEREST STATEMENT

The authors declare no conflicts of interest.

## ETHICS STATEMENT

This study was approved by the local ethic committee at GHU Paris Psychiatrie et Neurosciences, Sainte‐Anne Hospital.

## Supporting information


**Figure S1.** Summary of diffuse midline gliomas and posterior fossa group A ependymomas main characteristics. Astro., astrocytic; CPA, cerebellopontine angle; DMG, diffuse midline glioma; Oligo., oligodendroglial; PFA, posterior fossa group A ependymoma.

## Data Availability

Data sharing is not applicable to this article as no new data were created or analyzed in this study.
