# Supplementary figures and images for: The pontine diffuse midline glioma, EGFR‐subtype with ependymal features: Yet another face of diffuse midline glioma, H3K27‐altered
Source: Brain Pathol. 2023 Jun 18;34(1):e13181. doi: 10.1111/bpa.13181 (PMC10711257; doi:10.1111/bpa.13181)

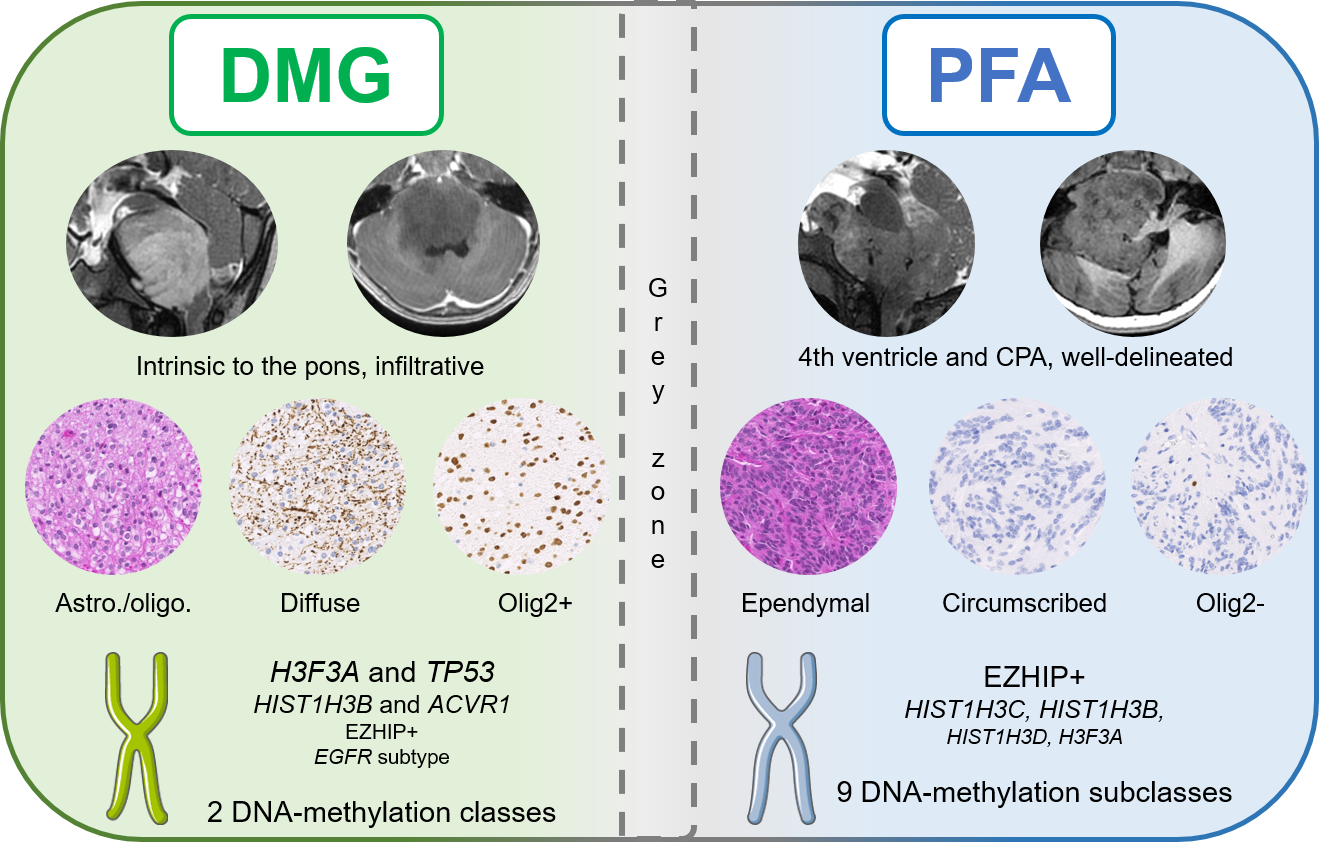

Supplement: Supplementary file 1 — Figure S1. Summary of diffuse midline gliomas and posterior fossa group A ependymomas main characteristics. Astro., astrocytic; CPA, cerebellopontine angle; DMG, diffuse midline glioma; Oligo., oligodendroglial; PFA, posterior fossa group A ependymoma. [file BPA-34-e13181-s001.tif]
